# Taxonomic resolution of different 16S rRNA variable regions varies strongly across plant-associated bacteria

**DOI:** 10.1093/ismeco/ycae034

**Published:** 2024-03-08

**Authors:** Katarina Hrovat, Bas E Dutilh, Marnix H Medema, Chrats Melkonian

**Affiliations:** Bioinformatics Group, Wageningen University and Research, 6708 PB Wageningen, The Netherlands; Theoretical Biology and Bioinformatics, Department of Biology, Faculty of Science, Utrecht University, 3584 CH Utrecht, The Netherlands; Institute of Biodiversity, Faculty of Biological Sciences, Friedrich Schiller University Jena, 07743 Jena, Germany; Cluster of Excellence Balance of the Microverse, Friedrich Schiller University Jena, 07745 Jena, Germany; Bioinformatics Group, Wageningen University and Research, 6708 PB Wageningen, The Netherlands; Bioinformatics Group, Wageningen University and Research, 6708 PB Wageningen, The Netherlands; Theoretical Biology and Bioinformatics, Department of Biology, Faculty of Science, Utrecht University, 3584 CH Utrecht, The Netherlands

**Keywords:** plant-microbiome, 16S metabarcoding analysis, phylogenomics, bioinformatics

## Abstract

Plant-microbiome research plays a pivotal role in understanding the relationships between plants and their associated microbial communities, with implications for agriculture and ecosystem dynamics. Metabarcoding analysis on variable regions of the 16S ribosomal RNA (rRNA) gene remains the dominant technology to study microbiome diversity in this field. However, the choice of the targeted variable region might affect the outcome of the microbiome studies. In our *in silico* analysis, we have evaluated whether the targeted variable region has an impact on taxonomic resolution in 16 plant-related microbial genera. Through a comparison of 16S rRNA gene variable regions with whole-genome data, our findings suggest that the V1–V3 region is generally a more suitable option than the widely used V3–V4 region for targeting microbiome analysis in plant-related genera. However, sole reliance on one region could introduce detection biases for specific genera. Thus, we are suggesting that while transitioning to full-length 16S rRNA gene and whole-genome sequencing for plant-microbiome analysis, the usage of genus-specific variable regions can achieve more precise taxonomic assignments. More broadly, our approach provides a blueprint to identify the most discriminating variable regions of the 16S rRNA gene for genera of interest.

## Introduction

Plant-associated microbes play important roles in supporting plant growth, health, and stress resistance. To comprehend how plants can benefit from their microbe partners, methods to accurately determine microbial taxonomy at species- and even strain-level resolution are essential [1]. This is because certain species or even strains within a species could perform different functions that either boost or reduce plant fitness [2].

The introduction of the 16S ribosomal RNA (rRNA) gene as a bacterial diversity marker was a breakthrough for microbial ecology studies [3]. Nowadays, analysis of 16S rRNA sequences is often used for the identification of bacterial taxonomy [4]. The 16S rRNA gene is ~1500 base pairs long and includes nine variable regions (V1–V9) [5]. Although sequencing the entire 16S rRNA gene would provide better taxonomic resolution [6], short-read sequencing analysis remains a cost-effective and widely used strategy for unraveling the microbiome composition across diverse environments [7]. The selection of specific 16S rRNA variable region(s) targeted by corresponding primer pairs significantly influences the estimation of taxonomic diversity. These regions vary in discriminatory power among microbes, and their variability is taxonomically dependent. Choosing the most suitable region remains an open question [6]. A comprehensive catalog of the most distinctive variable region for each taxonomic group is missing, underscoring the need for enhanced methodologies and analyses to address this gap. Furthermore, only few studies have explored the impact of 16S rRNA targeted regions on plant-associated microbes [8, 9].

To address the gap of knowledge on how 16S rRNA metabarcoding analysis discriminates the taxonomy within plant-associated microbes, we studied 16 of the most important plant-associated microbial genera. While most studies rely only on studying the genetic diversity within the 16S rRNA gene [6, 8-10], we used *in silico* analysis on complete, high quality genomes as the ground truth for our phylogenetic analyses (Table 1). To identify the most appropriate variable regions of 16S rRNA for microbiome analysis, we compared the phylogenies derived from the variable regions of 16S rRNA, the whole 16S rRNA gene, whole-genome average nucleotide identity (ANI) and sets of single-copy marker genes (SCMG). Phylogeny based on SCMGs is known for yielding robust and consistent phylogenetic trees [11] and ANI has been proposed as a new standard for defining microbial species [12]. Our approach integrates phylogeny across multiple levels of genomics information. It explores diversity within the 16S rRNA gene, providing a comprehensive analysis on selecting the most informative taxa-specific 16S rRNA variable region for plant-related microbiome research.

**Table 1:** Taxonomic overview of the analyzed plant-associated bacteria. Genomes were downloaded from the BV-BRC database and composed of complete and whole-genome sequencing genomes. Genera closely relatedto the selected plant-related genera are marked with^*^. Number of genomes represents the number of analyzed genomes after selection of genomes with good genome status and quality. Genomes were grouped into ANI groups using a 95% identity threshold.

**Phylum**	**Class**	**Order**	**Family**	**Genus**	**Number of genomes**	**Number of ANI groups**
Firmicutes	Bacilli	Bacillales	Bacillaceae	*Bacillus*	1363	126
				Paenibacillus^*^	146	83
Actinomycetota	Actinomycetia	Streptomycetales	Streptomycetaceae	*Streptomyces*	388	220
				Kitasatospora^*^	26	21
		Micromonosporales	Micromonosporaceae	*Actinoplanes*	18	16
				Micromonospora^*^	162	92
Pseudomonadota	Alphaproteobacteria	Azospirillales	Azospirillaceae	*Azospirillum*	32	22
				Nitrospirillum^*^	4	3
		Rhizobiales	Rhizobiaceae	*Ensifer*	22	14
				Sinorhizobium^*^	118	15
				*Mesorhizobium*	110	62
				Aminobacter^*^	21	9
				*Rhizobium*	231	119
				Agrobacterium^*^	91	20
			Xanthobacteraceae	*Bradyrhizobium*	223	79
				Rhodopseudomonas^*^	33	16
		Burkholderiales	Burkholderiaceae	*Massilia*	27	23
	Gammaproteobacteria			Duganella^*^	23	11
				*Cupriavidus*	93	31
				Ralstonia^*^	169	12
				*Burkholderia*	483	40
				Paraburkholderia^*^	167	79
		Enterobacterales	Enterobacteriaceae	*Serratia*	236	18
				Rahnella^*^	38	10
				*Enterobacter*	282	25
				Citrobacter^*^	359	19
		Pseudomonadales	Pseudomonadaceae	*Pseudomonas*	1350	291
				Azotobacter^*^	11	3
		Xanthomonadales	Xanthomonadaceae	*Xylella*	74	2
				*Xanthomonas*	422	27
				Pseudoxanthomonas^*^	99	42

## Results and discussion

**Figure 1:**
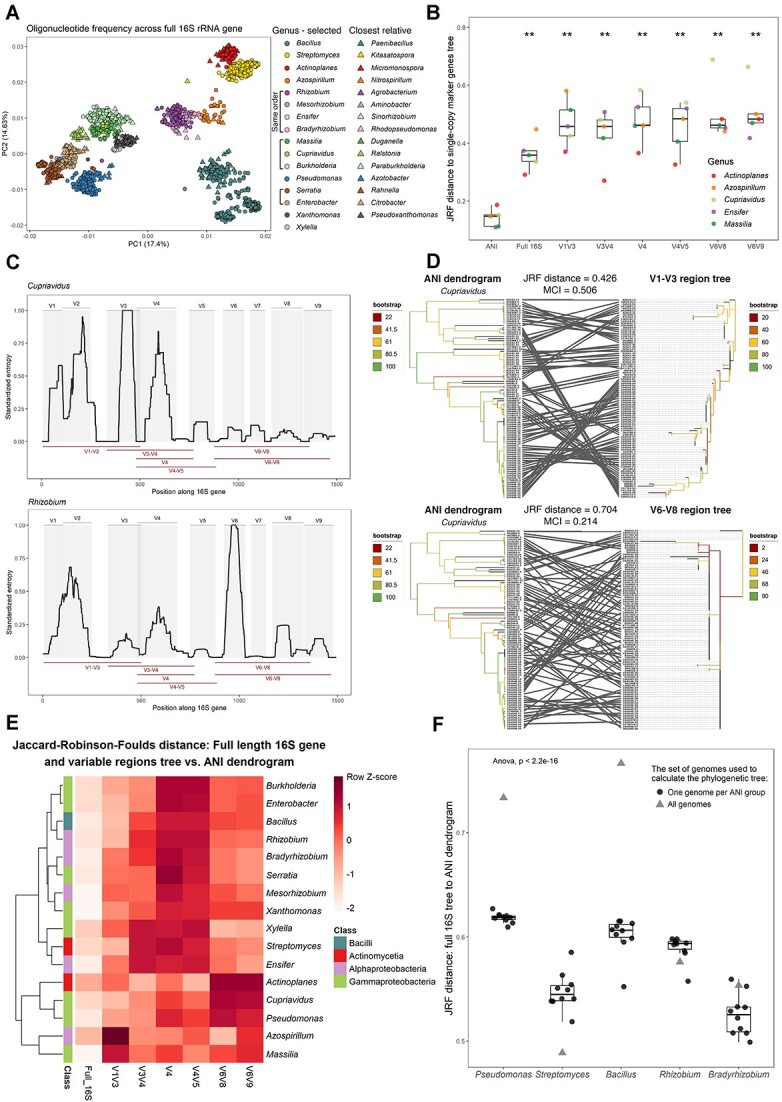
Identification of the most discriminating 16S rRNA gene regions. (A) A principal component analysis plot visualizes the oligonucleotide frequency patterns within full-length 16S rRNA gene sequences belonging to selected plant-related genera (represented as circles) and their closest relatives (represented as triangles) at the genus-level. Brackets indicate genera within the same order. (B) JRF distances between dendrogram of ANI and phylogenetic trees of full-length 16S gene and different variable regions to phylogenetic tree of SCMG. The line inside the boxplot represents the median, with the lowest and highest values within the 1.5 interquartile range represented by the whiskers. Significant differences between ANI and other variable regions are indicated with stars (Wilcoxon test, ^*^^*^: *P* ≤ .01). (C) Shannon entropy across the 16S rRNA gene based on the alignment of all selected 16S rRNA gene sequences within *Cupriavidus* genus (upper panel) and *Rhizobium* (lower panel). Gray panels show variable regions defined by commonly used primer-binding sites for plant-associated bacteria. Variable regions considered in this study are shown as red lines. (D) Comparison of *Cupriavidus* ANI dendrogram to phylogenetic tree using V1–V3 variable region (considered as the best region in E; top) and phylogenetic tree using V6–V8 variable region (considered as the worst region in E; bottom). Tree branches are colored according to the bootstrap values. Lines connecting the same strains of both trees aim at highlighting the degree of similarities between trees. The corresponding JRF distance and mutual clustering information (MCI) are presented on top of each tree comparisons. (E) The color scale depicts JRF distance *z*-score between phylogenetic trees of full length 16S rRNA tree and different variable regions of 16S rRNA gene to ANI dendrogram. Whiter cells signify a higher degree of similarity between the trees in question and the ANI dendrogram, whereas darker red cells indicate greater dissimilarity. Column annotations indicate the classification of specific genera into class as per the GTDB classification. (F) Boxplots showing JRF distance between phylogenetic tree constructed from full-length 16S tree and ANI dendrogram for all genomes in certain genera (gray triangles) and subset of selected genomes (black circles). The boxplots for each genus are arranged from left to right on the decreasing number of ANI groups. To reduce strain diversity, one genome from each ANI group was randomly selected from the entire genome collection per genus. This selection process was repeated 10 times.

Here we use 16S rRNA gene sequences from 6821 complete genomes of 16 most important genera of plant-related microbes that can be found in the rhizosphere, phyllosphere, or endosphere, based on our literature review (see Supplementary Methods, section Genome gathering). We gained first insights into the genomes in our data set and evaluated the effectiveness of distinguishing taxonomic groups through oligonucleotide frequency motifs. We included genomes of phylogenetically closely related genera and performed a Principal Component Analysis, which revealed that the majority of the selected genera seem to differentiate at the order-level (Fig. 1A). However, this analysis indicated that 16S rRNA gene motifs may not distinguish within-genus level taxonomy.

To better evaluate the diversity within genera of our genome collection, we constructed phylogenies based on SCMG, and ANI. Utilizing an ANI distance matrix, we computed a bootstrap-supported dendrogram. Hence, we integrated SCMG and ANI information as the phylogenetic ground truth to benchmark the most reliable taxon-specific variable region. Phylogenetic relationships inferred from SCMG were compared with the dendrogram of ANI and topology of trees obtained from analysis of full length 16S rRNA gene and its variable regions: V1–V3, V3–V4, V4, V4–V5, V6–V8, and V6–V9 (Fig. 1B). We used generalized Jaccard–Robinson–Foulds (JRF) distances to evaluate if the ANI dendrogram is more similar to the SCMG phylogeny than the 16S rRNA gene phylogeny (see Supplementary Methods, Fig. 1B). Based on this analysis, we can conclude that ANI information is a good representation of the SCMG phylogeny, with an average JRF distance of only 0.141 (Fig. 1B).

To better understand the variability across 16S rRNA gene among genera, we examined each genus individually. For example, the V1–V3 region offers the most accurate phylogenetic description for the *Cupriavidus* genus, but it performs less effectively for the *Massilia* genus (see Fig. 1B and E). To explain these variations, we used Shannon entropy to assess the degree of sequence variability across the 16S rRNA gene for each genus (Fig. 1C and Supplementary Fig. 1). In *Cupriavidus*, we observed the highest variability within the variable regions V1–V4, whereas the remaining sequence of the gene is highly conserved (Fig. 1C—top). Consequently, using the conserved V6–V8 region, the phylogenetic tree notably differed from the ANI dendrogram. The JRF distance was high (0.704), resulting in high dissimilarity between the examined trees (Fig. 1D—bottom). In contrast, employing the V1–V3 region yielded a phylogenetic tree with lower dissimilarity to the ANI dendrogram, with a JRF distance of 0.426 (Fig. 1D—top). We can conclude that the variation of variable regions differs between genera. This could be explained by taxon-specific evolution occurring over time through a nearly neutral selection [13]. Such mutations tend to cluster in “hot spots,” which differ between species [14]. Recognizing and leveraging these differences is crucial for microbiome studies.

In Fig. 1E, we provide a comprehensive comparison of the variable regions for each genus. As expected, the full-length 16S rRNA gene demonstrated overall the best performance. Regarding the variable regions, the V1–V3 region demonstrated the best results for half of the analyzed genera and V6–V9 for four genera. Opposite, the V4 region alone could not successfully distinguish genomes in any of the genera. V6–V8 and V3–V4 demonstrated the best results for two and one genus, respectively. Notably, in genera like *Xylella*, *Massilia*, *Ensifer*, *Azospirillum*, and *Actinoplanes*, using the V1–V3 region may not give the best results, as other regions like V6–V8 and V6–V9 outperformed it (see Fig. 1E). The V3–V4 variable region, often considered as the gold standard for taxonomic identification in microbiome analysis [15, 16], exhibits the highest resolving power only in *Actinoplanes*. Interestingly, we observed that the phylogenetic relationships between the genera are not a good indicator to select a common best variable region for microbiome analysis. This is evident in the low homogeneity (0.214) and completeness (0.198) between the clustering in the dendrogram of the heatmap (Fig. 1E) and the expected clustering into four bacteria classes.

Understanding ecosystem dynamics in plant microbiome studies requires considering the variability within microbial species [17]. Given the varying availability of genomes across species and genera, we removed the strain variability to assess its effect on tree-to-tree distances using single representatives per species, as follows. We selected one genome per ANI group (see Supplementary Methods) and examined how this affected the JRF distance between the full-length 16S rRNA tree and the ANI dendrogram (Fig. 1F). For *Bacillus* and *Pseudomonas*, with the highest strain diversity in our collection, the JRF distance decreased from 0.77 to an average of 0.6 and from 0.73 to an average of 0.55, respectively. In contrast, in genera with lower strain diversity, reducing variability had little impact on the distance. Using a single genome per ANI group for the entire 16S rRNA tree resulted in a high average JRF distance (0.57). The observed variation in distances between phylogenetic trees, where we selected one genome per ANI group to the ANI dendrogram, showed statistically significant differences among genera (*P*-value < .001). Additionally, the distance was correlated with the number of ANI groups per genera (*P* = .0011, *R* = 0.45, Supplementary Fig. 2). Overall, these results highlight the limitations of even the full-length 16S rRNA tree in discriminating between strains and species within these genera.

In this study, we compared phylogenetic trees built from variable regions to the genome-wide ANI dendrogram to determine the variable region of 16S rRNA gene that provided the best taxonomic resolution in plant microbiome studies. The recently developed long-read sequencing of the full-length 16S rRNA gene and metagenomes offers better results on taxonomy discrimination, as confirmed in this study specifically for the full-length 16S rRNA gene. However, sequencing variable regions remains a cost-effective option when a microbiome profile at the genus rank or higher is sufficient. The presented results offer a means to assess microbiome findings on species- and strain-level resolution related to the chosen plant-associated bacteria genera. Furthermore, our methodology, which uses whole-genome information, could be utilized for identifying and assessing alternative marker genes [18, 19]. More importantly, by comparing phylogenies from 16S rRNA gene variable regions to whole-genome data, we found that V1–V3 stood out as a generally reliable choice for plant-related genera [20]. Caution is advised as relying solely on a variable region may introduce detection biases for specific genera. Therefore, depending on the objective of the research, a careful selection of the variable region is needed in order for 16S rRNA metabarcoding analysis to remain relevant in future microbiome research.

## Supplementary Material

Supplementary_material_ycae034

## Data Availability

The genome IDs used in this analysis, along with instructions on how to download them, are available on our GitHub repository: https://github.com/hrovatkatarina/How_good_is_16S_gene.git. Please refer to the README page of the repository for detailed information on accessing the genomes.

## Code availability

A copy of the code used for the analyses reported in this manuscript can be found at: https://github. com/hrovatkatarina/How_good_is_16S_gene.git.
