# Supplementary material for: Taxonomic resolution of different 16S rRNA variable regions varies strongly across plant-associated bacteria
Source: ISME Commun. 2024 Mar 8;4(1):ycae034. doi: 10.1093/ismeco/ycae034 (PMC10980831; doi:10.1093/ismeco/ycae034)
Supplement: Supplementary_material_ycae034 [file supplementary_material_ycae034.pdf]

Taxonomic resolution of different 16S rRNA variable regions varies strongly across plant-associated bacteria

Katarina Hrovat, Bas E. Dutilh, Marnix H. Medema, and Chrats Melkonian

Supplementary information

Supplementary Methods

Genome gathering

We selected 16 the most important genera that form interactions with plants based on the comprehensive literature review. We selected the most important genera of rhizobia[1,2], plant-growth promoting bacteria[3,4] and phytopathogenic bacteria[5]. In addition we consulted domain-specific experts within the Microbial Imprinting for Crop Resilience (MiCRop) consortium. After the selection of genera we chose their close relatives based on phylogeny using AnnoTree[6], which provides both phylogenetic and taxonomy information derived from the GTDB database[7]. We further selected genera that were phylogenetically closest to the 16 selected genera and had the highest number of genomes available in the database (Table 1). Additionally, we considered that the selected closely related genera are related to plants. This information was assessed from BV-BRC database[8], where the Host Common Name of selected genomes was specified as a plant. All genomes used in our study were retrieved from BV-BRC database[8], and composed of complete and whole-genome sequencing (WGS) genomes. We selected genomes where genome quality described in BV-BRC database was categorized as 'good'. The WGS genomes were selected based on the following criteria: greater than 90% genome CheckM completeness, less than 10% genome CheckM contamination, and fewer than 100 contigs. We continued our analysis with a total of 6821 genomes. Average Nucleotide Identity (ANI) was computed with ANIclustermap[9], where ANI between all-vs-all genomes was calculated by fastANI tool[10]. Genomes were then grouped into ANI groups, using a 95% identity threshold. Note: We observed discrepancies in the taxonomic annotation of the genomes of the *Ensifer* and *Sinorhizobium* genus in our database since after ANI calculation, some genomes annotated as different genus were considered as same species. This is only relevant for PCA plot (Figure 1a) and does not affect further analysis.

16S rRNA gene based phylogeny

16S rRNA gene sequences were identified by predicting the location by Barrnap (Version 0.8). In case where more than one copy of 16S rRNA sequence was identified, we selected the copy with the longest sequence. The lengths of the sequences were inspected to ensure they fell within the acceptable range of 1500 bp. If the identified 16S sequence was shorter, the entire genome was excluded. These 16S rRNA sequences were aligned by ssu-align[11] and than grouped by genera. Shannon entropy was calculated at each base position for each analyzed genus individually using the formula: $H = -\sum_i (p(i) \log_2(p(i)))$. We standardized the entropy values and applied a moving average filter with a window size of 70 to enhance the smoothness of the entropy data. For visualization (Figure 1c, Supplementary Figure 1a-n), we utilized the code provided in the repository of Johnson et al. with some modifications[12]. Oligonucleotide frequency of full-length 16S rRNA sequences were calculated using oligonucloetideFrequency function of the Biostrings R package (version 2.68.1), with 4 nucleotides per oligonucleotide (width) and the sliding window step of 1. PCA was performed using the prcomp function of the stats R package (version 4.3.0). Data was zero-centered and scaled, so the variables have unit variance before the analysis took place.

Variable regions (V1-V3, V3-V4, V4, V4-V5, V6-V8 and V6-V9) were determined based on primer pairs (Supplementary Table 1) commonly used in soil-microbiome studies[13]. In our analysis we also included V4 and V6-V9 region, so the sequences of V4 forward and V9 reverse primers were obtained from Johnson et al.[12]

Single-copy marker genes based phylogeny

In order to identify single-copy marker genes we selected genera with low number of genomes (*Actinoplanes*, *Azospirillum*, *Cupriavidus*, *Ensifer* and *Massilia*), as we could not obtain an alignment due to the large number of genomes. The genes were first annotated using Prokka[14]. Alignments of single-copy

marker genes were generated by Roary[15], where we used the option for fast core gene alignment with MAFFT. A minimum percentage identity parameter was set at 75% and the maximum number of clusters was set to 70,000 for *Azospirillum* and *Cupriavidus* and to 58,000 for *Massilia* genus since at default settings (maximum 50,000 clusters) Roary didn't create the core alignment. For *Actinoplanes* and *Ensifer* default settings for the maximum number of clusters were retained.

Construction of phylogenetic trees

The alignments of full length 16S rRNA, different combination of variable regions and single-copy marker genes DNA sequences were utilized to generate phylogenetic trees conducted with the maximum likelihood method using the IQ-TREE tool with 1,000 bootstrap replicates[16]. The ANI distance matrix was used to build a bootstrap supported dendrogram by pvclust tool with 1,000 bootstrap replicates[17].

Distance calculations

The single-copy marker genes and the dendrogram of ANI were compared to full-length 16S rRNA gene and its variable region, utilizing the generalized Robinson-Foulds metrics. Jaccard-Robinson-Foulds distance[18,19] and the information-based measures of Mutual Clustering Information[20] were computed with the R package TreeDist (version 2.6.1). This metric identifies the optimal pairing by matching splits in one tree with corresponding splits in the other tree. The matchings are scored based on the size of the largest split that is consistent with both trees, and the score is then normalized against the Jaccard index. Similarity scores are normalized, so the results are rescaled from zero to one by dividing by the maximum possible value for trees of the given topologies, which is equal to the sum of the number of splits in each tree. Graphical comparison of trees was done using tanglegram function of dendextend R package (version 1.17.1). Visualization was done using iTOL[21].

We tested the significant differences between the distances of the ANI dendrogram and the phylogenetic trees of variable regions to the core-genome tree (Figure 1b). This comparison was conducted using the Wilcoxon test implemented through the stat_compare_means function from the ggpubr package (version 0.6.0), with a significance threshold (p-value) set at 0.05.

We assessed the accuracy of clustering bacterial genera by class based on the JRF distance of the phylogenetic trees of different variable regions compared to the ANI dendrogram. We divided the dendrogram in the heatmap (Figure 1e) into four groups using the cutree function from the stats package (version 4.3.0), as we anticipated grouping into four distinct classes. We calculated the cluster homogeneity and completeness between predicted and ground truth clustering using functions from the clevr package (version 0.1.2) .

Removing strain variability

To examine how removing strain variability impacted the distance between the full-length 16S rRNA tree and the ANI dendrogram (Figure 1f), we randomly selected one genome per ANI group and repeated the process 10 times for each genus. If there was a significant difference in distances between genera, it was tested with ANOVA using the aov function from the stats package (version 4.3.0). We used the Pearson correlation coefficient to assess the correlation between the number of ANI groups and the distance, employing the cor.test function from the stats package (version 4.3.0).

Supplementary Table and Figures

Table 1. Primers used to identify variable regions within 16S rRNA gene sequences.

16S Variable Region	Forward Sequence	Reverse Sequence
V1V3	AGAGTTTGATCMTGGCTCAG	TTACCGCGGCKGCTGGCACG
V3V4	CCTACGGGNGGCWGCAG	GACTACHVGGGTATCTAATCC
V4	GTGYCAGCMGCCGCGGTAA	GACTACHVGGGTATCTAATCC
V4V5	GTGYCAGCMGCCGCGGTAA	CCGYCAATTYMTTTRAGTTT
V6V8	AATTGACGGGGRCCCGC	ACGGGCRGTGWGTRCAA
V6V9	AATTGACGGGGRCCCGC	TACGGYTACCTTGTTAYGACTT

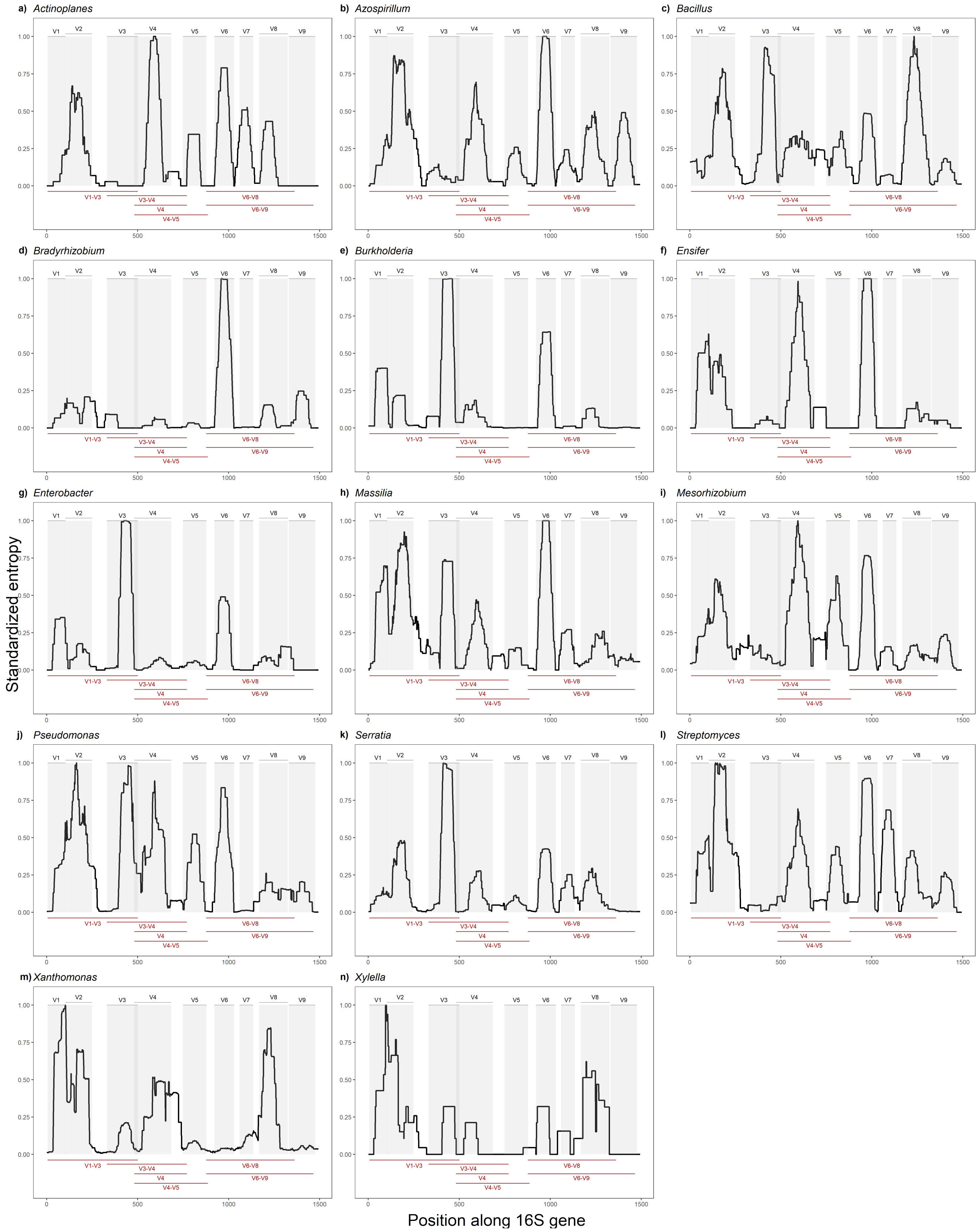

Supplementary Figure 1. Shannon entropy across the 16S rRNA gene based on the alignment of all selected 16S rRNA gene sequences within all selected genera (a-n). Gray panels show variable regions defined by commonly used primer-binding sites for soil bacteria. Variable regions considered in this study are shown as red lines.

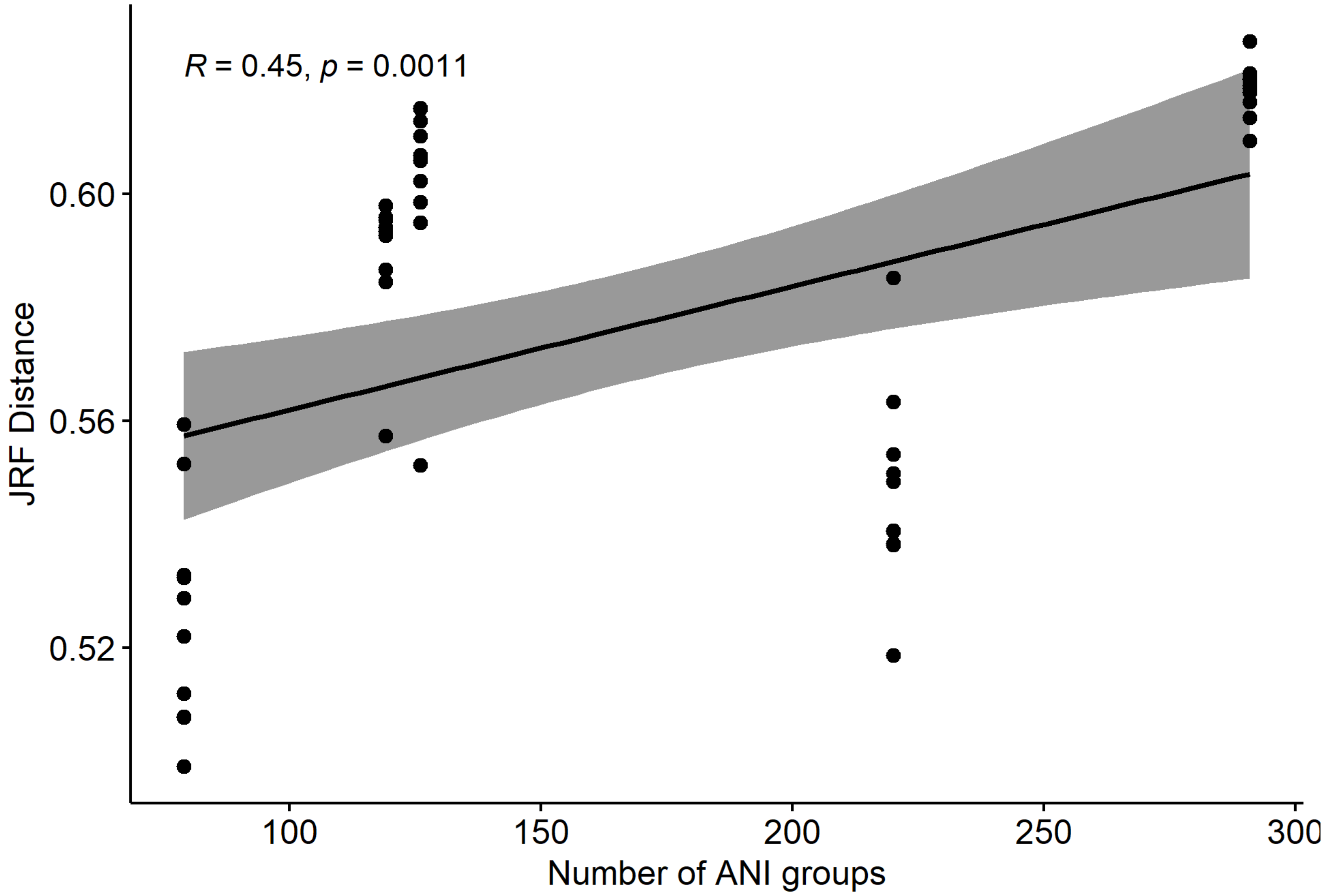

Supplementary Figure 2. Pearson correlation between Jaccard-Robinson-Foulds (JRF) distance, calculated in Figure 1f and the number of ANI groups.

References

1. Poole, P., Ramachandran, V. & Terpolilli, J. Rhizobia: from saprophytes to endosymbionts. *Nat. Rev. Microbiol.* **16**, 291–303, DOI: 10.1038/nrmicro.2017.171 (2018).
2. Seshadri, R. *et al.* Discovery of novel plant interaction determinants from the genomes of 163 root nodule bacteria. *Sci. Reports* **5**, DOI: 10.1038/srep16825 (2015).
3. Shukla, A. K. *Ecology and Diversity of Plant Growth Promoting Rhizobacteria in Agricultural Landscape*, 1–15 (Elsevier, 2019).
4. Katsenios, N. *et al.* Assessment of plant growth promoting bacteria strains on growth, yield and quality of sweet corn. *Sci. Reports* **12**, DOI: 10.1038/s41598-022-16044-2 (2022).
5. Martins, P. M. M., Merfa, M. V., Takita, M. A. & De Souza, A. A. Persistence in phytopathogenic bacteria: Do we know enough? *Front. Microbiol.* **9**, DOI: 10.3389/fmicb.2018.01099 (2018).
6. Mendler, K. *et al.* AnnoTree: visualization and exploration of a functionally annotated microbial tree of life. *Nucleic Acids Res.* **47**, 4442–4448, DOI: 10.1093/nar/gkz246 (2019).
7. Parks, D. H. *et al.* GTDB: an ongoing census of bacterial and archaeal diversity through a phylogenetically consistent, rank normalized and complete genome-based taxonomy. *Nucleic Acids Res.* **50**, D785–D794, DOI: 10.1093/nar/gkab776 (2021).
8. Olson, R. D. *et al.* Introducing the bacterial and viral bioinformatics resource center (BV-BRC): a resource combining PATRIC, IRD and ViPR. *Nucleic Acids Res.* **51**, D678–D689, DOI: 10.1093/nar/gkac1003 (2022).
9. Shimoyama, Y. ANIclustermap: A tool for drawing ANI clustermap between all-vs-all microbial genomes (2022).
10. Jain, C., Rodriguez-R, L. M., Phillippy, A. M., Konstantinidis, K. T. & Aluru, S. High throughput ANI analysis of 90k prokaryotic genomes reveals clear species boundaries. *Nat. Commun.* **9**, DOI: 10.1038/s41467-018-07641-9 (2018).
11. Nawrocki, E. P., Kolbe, D. L. & Eddy, S. R. Infernal 1.0: inference of RNA alignments. *Bioinformatics* **25**, 1335–1337, DOI: 10.1093/bioinformatics/btp157 (2009).
12. Johnson, J. S. *et al.* Evaluation of 16s rRNA gene sequencing for species and strain-level microbiome analysis. *Nat. Commun.* **10**, DOI: 10.1038/s41467-019-13036-1 (2019).
13. Soriano-Lerma, A. *et al.* Influence of 16s rRNA target region on the outcome of microbiome studies in soil and saliva samples. *Sci. Reports* **10**, DOI: 10.1038/s41598-020-70141-8 (2020).
14. Seemann, T. Prokka: rapid prokaryotic genome annotation. *Bioinformatics* **30**, 2068–2069, DOI: 10.1093/bioinformatics/btu153 (2014).
15. Page, A. J. *et al.* Roary: rapid large-scale prokaryote pan genome analysis. *Bioinformatics* **31**, 3691–3693, DOI: 10.1093/bioinformatics/btv421 (2015).
16. Minh, B. Q. *et al.* IQ-TREE 2: New models and efficient methods for phylogenetic inference in the genomic era. *Mol. Biol. Evol.* **37**, 1530–1534, DOI: 10.1093/molbev/msaa015 (2020).
17. Suzuki, R. & Shimodaira, H. Pvclust: an r package for assessing the uncertainty in hierarchical clustering. *Bioinformatics* **22**, 1540–1542, DOI: 10.1093/bioinformatics/btl117 (2006).
18. Nye, T. M., Liò, P. & Gilks, W. R. A novel algorithm and web-based tool for comparing two alternative phylogenetic trees. *Bioinformatics* **22**, 117–119, DOI: 10.1093/bioinformatics/bti720 (2005).

19. Böcker, S., Canzar, S. & Klau, G. W. The generalized robinson-foulds metric. In *Lecture Notes in Computer Science*, 156–169, DOI: 10.1007/978-3-642-40453-5_13 (Springer Berlin Heidelberg, 2013).

20. Smith, M. R. Information theoretic generalized robinson–foulds metrics for comparing phylogenetic trees. *Bioinformatics* **37**, 2077–2078, DOI: 10.1093/bioinformatics/btab200 (2021).

21. Letunic, I. & Bork, P. Interactive tree of life (itol) v5: an online tool for phylogenetic tree display and annotation. *Nucleic Acids Res.* **49**, W293–W296, DOI: 10.1093/nar/gkab301 (2021).
